# Characterization of Distinctive In Vivo Metabolism between Enhancing and Non-Enhancing Gliomas Using Hyperpolarized Carbon-13 MRI

**DOI:** 10.3390/metabo11080504

**Published:** 2021-07-31

**Authors:** Seunggwi Park, Hashizume Rintaro, Seul Kee Kim, Ilwoo Park

**Affiliations:** 1 Department of Radiology, Chonnam National University Medical School and Hwasun Hospital, Hwasun 58128, Korea; seunggwi1995@naver.com (S.P.); kimsk.rad@gmail.com (S.K.K.); 2 Department of Neurological Surgery and Biochemistry and Molecular Genetics, Northwestern University, Chicago, IL 60611, USA; rintaro.hashizume@northwestern.edu; 3 Department of Radiology, Chonnam National University Medical School and Hospital, Gwangju 61469, Korea; 4 Department of Artificial Intelligence Convergence, Chonnam National University, Gwangju 61186, Korea

**Keywords:** hyperpolarized ^13^C MRI, glioma, enhancing tumor and non-enhancing tumor, metabolites, brain tumor

## Abstract

The development of hyperpolarized carbon-13 (^13^C) metabolic MRI has enabled the sensitive and noninvasive assessment of real-time in vivo metabolism in tumors. Although several studies have explored the feasibility of using hyperpolarized ^13^C metabolic imaging for neuro-oncology applications, most of these studies utilized high-grade enhancing tumors, and little is known about hyperpolarized ^13^C metabolic features of a non-enhancing tumor. In this study, ^13^C MR spectroscopic imaging with hyperpolarized [1-^13^C]pyruvate was applied for the differential characterization of metabolic profiles between enhancing and non-enhancing gliomas using rodent models of glioblastoma and a diffuse midline glioma. Distinct metabolic profiles were found between the enhancing and non-enhancing tumors, as well as their contralateral normal-appearing brain tissues. The preliminary results from this study suggest that the characterization of metabolic patterns from hyperpolarized ^13^C imaging between non-enhancing and enhancing tumors may be beneficial not only for understanding distinct metabolic features between the two lesions, but also for providing a basis for understanding ^13^C metabolic processes in ongoing clinical trials with neuro-oncology patients using this technology.

## 1. Introduction

Gliomas are the most common primary brain tumor in adults [1]. According to the World Health Organization (WHO) classification, gliomas are pathologically classified into subtypes, such as astrocytoma, oligodendroglioma, oligoastrocytoma, and glioblastoma, which are graded into WHO grade I to IV according to the degree of malignancy [2]. A diffuse midline glioma is a specific entity of glioma based on H3 K27M mutation [2]. Gliomas are infiltrative in nature, and have a limited sensitivity to radiation and chemotherapy; therefore, the prognosis for patients with gliomas is poor. Treatment for gliomas is complex, which consists of surgical resection, radiation therapy, and chemotherapy. The current clinical standard for overseeing the response of the glioma to treatment is clinical symptoms and imaging evaluation using magnetic resonance imaging (MRI).

The Macdonald Criteria, which uses a contrast-enhancing component on MRI in conjunction with clinical assessment and a corticosteroid dose, had been initially used for evaluating tumor response in clinical trials of high-grade gliomas [3]. Revised Assessment in Neuro-Oncology (RANO) criteria was published in 2010, which included an added focus on using T2/fluid-attenuated inversion recovery (FLAIR) for response assessment [4]. The T2/FLAIR assessment resulted in earlier detection of progression in a subgroup of patients, with at least 35% of patients showing an increase in non-enhancing tumors, which would not have met progression standards under the Macdonald Criteria [3,4]. Since the introduction of RANO for glioma response evaluation, there has been growing interest in understanding the features that characterize and distinguish non-enhancing tumors from other processes [5,6]. Although various advanced MRI modalities beyond conventional MR imaging, such as diffusion-weighted imaging, perfusion-weighted imaging, and MR spectroscopy, have been previously used for identifying and comparing physiological properties between enhancing and non-enhancing tumors [7,8], the characterization of the molecular and biochemical properties of these lesions is still a challenging endeavor. 

Hyperpolarized carbon-13 (^13^C) MRI is a new imaging platform that has made the real-time imaging of in vivo metabolism possible, with a high signal-to-noise ratio (SNR) and a short scan time. This technique has been applied to pre-clinical and clinical studies of brain tumors [9,10,11,12,13,14,15,16]. Although previous studies have demonstrated the utility of this technique for distinguishing cancerous tissue from normal tissue and monitoring the response to therapy in a brain tumor, most of these studies have used high-grade brain tumor models with an enhancing lesion. Very little is known regarding the in vivo hyperpolarized metabolism of a non-enhancing tumor and how it is compared to that of an enhancing tumor. 

The purpose of this study was to evaluate the feasibility of probing and comparing distinctive in vivo metabolism between enhancing and non-enhancing brain tumors using hyperpolarized ^13^C MR spectroscopic imaging (MRSI). We used [1-^13^C]pyruvate as a metabolic substrate, which is the most widely utilized compound in hyperpolarized ^13^C cancer imaging. The rat models of enhancing and non-enhancing tumors were created by the intracranial injection of glioblastoma (U87-MG) and diffuse midline glioma (SF8628) cell lines, respectively.

## 2. Results

**Figure 1:**
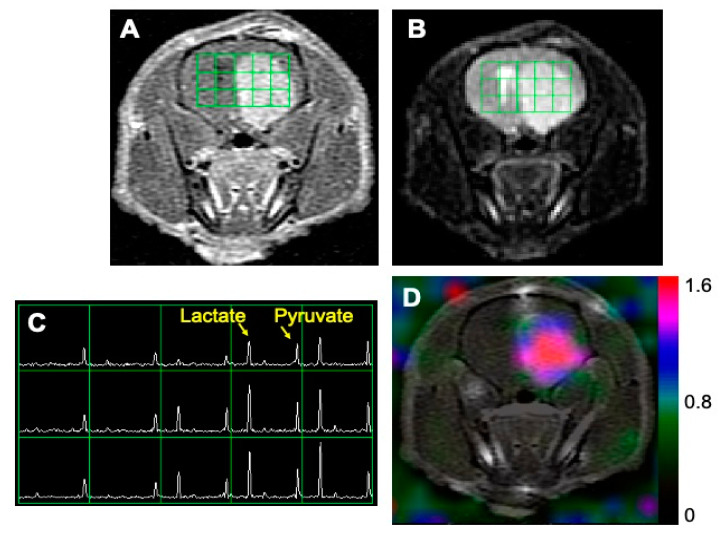
An example of MR imaging data from a rat model of glioblastoma. A post-gadolinium T1-weighted image (**A**) and T2-weighted image (**B**) showed the typical features of a high-grade glioma: contrast enhancement in the post-contrast T1-weighted image and hyperintensity in the T2-weighted image. The grid in (**A**,**B**) represents the array of ^13^C spectroscopic imaging data over the brain, with an in-plane voxel resolution of 2 × 2 mm (**C**). Each voxel in (**C**) contains the hyperpolarized ^13^C pyruvate signal and its metabolic product, lactate. The color overlay map of the ratio of lactate to pyruvate in (**D**) shows a highly elevated level of lactate in the enhancing lesion.

Figure 1 shows an example of ^1^H and ^13^C imaging data from a rat model of glioblastoma. The post-gadolinium (Gd) T1-weighted image (T1WI, Figure 1A) and T2-weighted image (T2WI, Figure 1B) showed typical morphological features of high-grade gliomas: a contrast-enhancing lesion in post-contrast T1WI and hyperintensity in T2WI. The green grids on top of T1WI and T2WI represented the array of ^13^C metabolic data over the brain, with an in-plane voxel resolution of 2 × 2 mm, which corresponded to the MRSI spectra in Figure 1C. Each voxel in Figure 1C contains the hyperpolarized ^13^C pyruvate signal and its metabolic product, lactate. Figure 1D is a color overlay map of the ratio of lactate to pyruvate (Lac/Pyr), which shows a high level of Lac/Pyr in the enhancing lesion.

**Figure 2:**
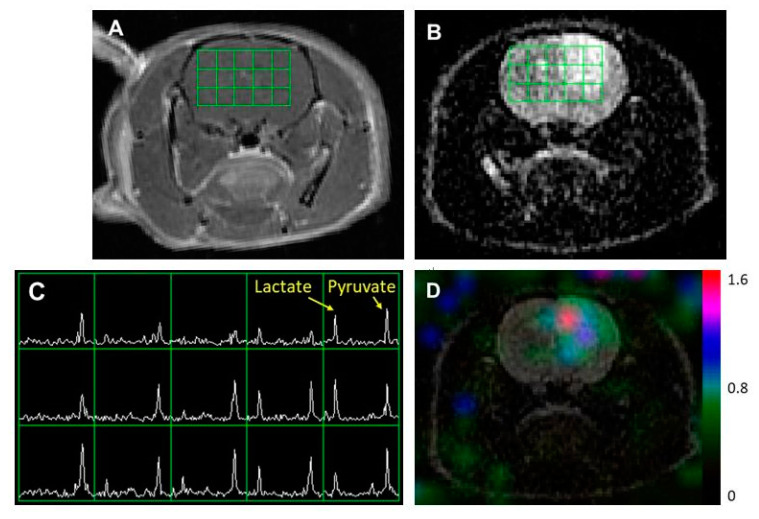
An example of MR imaging data from a rat model of a diffuse midline glioma. A post-Gd T1-weighted image (**A**) and T2-weighted image (**B**) showing the typical features of a non-enhancing brain tumor: hyperintensity in the T2-weighted image without contrast enhancement. The grid in (**A**,**B**) represents the array of ^13^C spectroscopic imaging data over the brain (**C**) with an in-plane voxel resolution of 2 × 2 mm, showing hyperpolarized metabolite signals from pyruvate and lactate. The color overlay map of the ratio of lactate to pyruvate in (**D**) shows an elevated level of lactate in the non-enhancing lesion compared to the surrounding tissue.

Figure 2 illustrates representative ^1^H and ^13^C imaging data from a rat model of a diffuse midline glioma. The post-Gd T1WI (Figure 2A) and T2WI (Figure 2B) exhibited the typical radiographical features of a non-enhancing brain tumor: hyperintensity in T2WI without contrast enhancement. Similar to the glioblastoma model with an enhancing tumor, the region with hyperintensity exhibited an elevated level of lactate compared to the surrounding brain tissue. 

**Figure 3:**
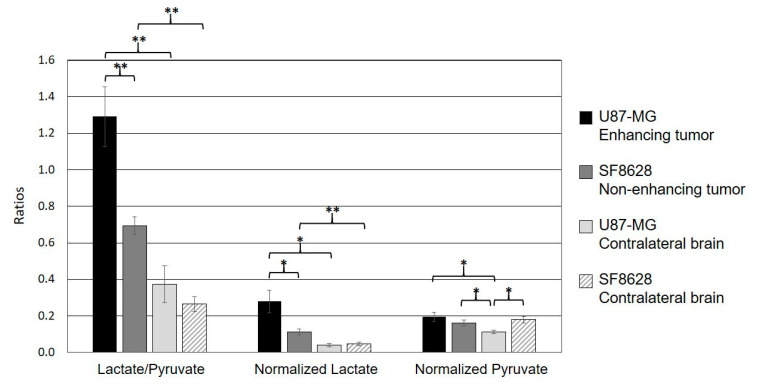
The comparison of quantified metabolic parameters from the two types of tumor models. Both the ratio of lactate to pyruvate (Lactate/Pyruvate) and normalized lactate in the enhancing lesion of the U87-MG models were significantly higher than those in the T2 hyperintense lesion of the SF8628 models. The levels of normalized pyruvate were comparable between the two types of tumor. In both types of tumor, the levels of Lac/Pyr and normalized lactate were significantly higher than those in contralateral normal-appearing brain tissue. The level of normalized pyruvate in the contralateral brain tissue of the rats bearing glioblastoma was significantly smaller than that in its enhancing lesion, which was comparable to the level of normalized pyruvate in the non-enhancing lesion and the contralateral brain tissue of the rats bearing a diffuse midline glioma. In contrast, the rats bearing a diffuse midline glioma displayed a comparable level of normalized pyruvate between its ipsilateral hyperintense lesion and the contralateral normal tissue (* *p* < 0.05; ** p < 0.01). Error bars indicate the standard error of the mean.

Table 1 and Figure 3 show the quantified ^13^C metabolites from the two types of tumor models. Lac/Pyr, normalized lactate, and pyruvate were evaluated using the voxels containing contrast enhancing lesions from rats bearing glioblastoma and non-enhancing hyperintensity from rats bearing a diffuse midline glioma. The contralateral normal appearing brain tissues from each type of tumor were also evaluated. The enhancing tumor exhibited a higher level of pyruvate-to-lactate conversion than the non-enhancing tumor. Both Lac/Pyr (1.29 ± 0.16) and normalized lactate (0.28 ± 0.06) in the enhancing lesion of U87-MG rats were significantly higher than those in the non-enhancing lesion of SF8628 rats (Lac/Pyr, 0.69 ± 0.05, *p* = 0.0041; normalized lactate, 0.11 ± 0.02, *p* = 0.020). The levels of normalized pyruvate were comparable between the two types of tumors (0.20 ± 0.02 in U87-MG vs. 0.16 ± 0.02 in SF8628, *p* = 0.24). While the rats bearing enhancing and non-enhancing tumors possessed similar levels of both Lac/Pyr (0.37 ± 0.10 in U87-MG vs. 0.26 ± 0.04 in SF8628, *p* = 0.31) and normalized lactate (0.04 ± 0.01 in U87-MG vs. 0.05 ± 0.01 in SF8628, *p* = 0.69) in their contralateral normal-appearing brain tissues, the level of normalized pyruvate in the contralateral normal-appearing brain tissue of rats bearing non-enhancing lesions (0.18 ± 0.02) was significantly higher than that of rats bearing enhancing lesions (0.11 ± 0.01, *p* = 0.014).

When the ^13^C metabolite parameters in the ipsilateral lesion and contralateral normal brain tissue were compared, the rats bearing glioblastoma exhibited significantly higher levels of both Lac/Pyr (1.29 ± 0.16) and normalized lactate (0.28 ± 0.06) in the enhancing tumor compared to the contralateral normal-appearing brain tissue (Lac/Pyr, 0.37 ± 0.10, *p* = 0.00065; normalized lactate, 0.04 ± 0.01, *p* = 0.013). Similarly, the levels of both Lac/Pyr (0.69 ± 0.05) and normalized lactate (0.11 ± 0.02) in the non-enhancing tumor from rats bearing a diffuse midline glioma were significantly higher than those in the corresponding contralateral normal brain tissue (Lac/Pyr, 0.26 ± 0.04, *p* = 0.00013; normalized lactate, 0.05 ± 0.01, *p* = 0.0018). In a comparison of pyruvate between an ipsilateral tumor and a contralateral normal brain, the rats bearing glioblastoma exhibited a significantly higher level of normalized pyruvate in the enhancing lesion (0.20 ± 0.02) compared to the contralateral normal tissue (0.11 ± 0.01, *p* = 0.033). In contrast, the rats bearing a diffuse midline glioma displayed a comparable level of normalized pyruvate between the ipsilateral hyperintensity (0.16 ± 0.02) and the contralateral normal tissue (0.18 ± 0.02, *p* = 0.27). 

## 3. Discussion

Imaging modalities play an important role in the management of high-grade gliomas. The analysis of imaging findings for diagnosis and monitoring of the response to therapy after surgery, radiation, and chemotherapy requires the understanding of morphologic as well as biochemical changes in both enhancing and non-enhancing components of a tumor. The current study demonstrated the feasibility of using hyperpolarized ^13^C MR metabolic imaging to characterize and compare distinct metabolic patterns in enhancing and non-enhancing brain tumors using rodent models of glioblastoma and diffuse midline gliomas. 

Both the enhancing and non-enhancing glioma models exhibited significantly higher levels of Lac/Pyr and normalized lactate in their ipsilateral tumors compared to the corresponding contralateral normal-appearing brain tissue. The elevated level of lactate in both tumors conforms with the typical feature of cancer, known as the Warburg effect [17], which explains elevated aerobic glycolysis and the consequent increase in lactate production. The enhancing tumor demonstrated a significantly higher level of both Lac/Pyr and normalized lactate compared to the non-enhancing tumor. The levels of normalized pyruvate in the enhancing and non-enhancing tumors were comparable, indicating that the amount of pyruvate delivered and taken up by the two types of tumor was comparable, and substrate availability was not the reason for the difference in the level of lactate between the enhancing and non-enhancing tumors. Although it is beyond the scope of this study to elucidate the explicit reason for the difference in the level of lactate between the enhancing and non-enhancing tumors, there may exist inherent molecular differences that resulted in distinct metabolic profiles between the two types of tumor. Previous evidence has indicated that the increased labeling of lactate correlates with tumor grade in a pre-clinical model of prostate cancer [18]. Several clinical studies also have shown that the elevated level of lactate correlates with increased tumor Gleason grade in prostate cancer [19], higher grade breast cancer [20], such as triple-negative breast cancer, and progressive gliomas following anti-angiogenic therapy [9]. These studies, along with our results, highlight the potential of hyperpolarized ^13^C metabolic imaging for the non-invasive subtyping of tumors based on the assessment of pyruvate utilization. 

It is noteworthy that the enhancing tumor showed a significantly higher level of normalized pyruvate than its contralateral normal-appearing tissue, while the levels of pyruvate in the ipsilateral and contralateral tissues in the non-enhancing tumor were similar. In Gd-enhanced MRI, the enhancement of brain tumors is caused by blood–brain barrier disruption and the subsequent contrast leakage from the vasculature into the tumor and surrounding tissue. In the contrast-enhancing tumor, a large amount of pyruvate was most likely permeable to the tumor and surrounding tissue through the disrupted blood–brain barrier. This resulted in a significantly higher level of normalized pyruvate in the enhancing tumor compared to its contralateral brain tissue, which presumably maintained a relatively intact blood–brain barrier. In contrast, the non-enhancing tumor supposedly possessed a relatively undamaged blood–brain barrier; hence, the level of normalized pyruvate was similar between the non-enhancing tumor and its contralateral normal-appearing brain tissue.

**Figure 4:**
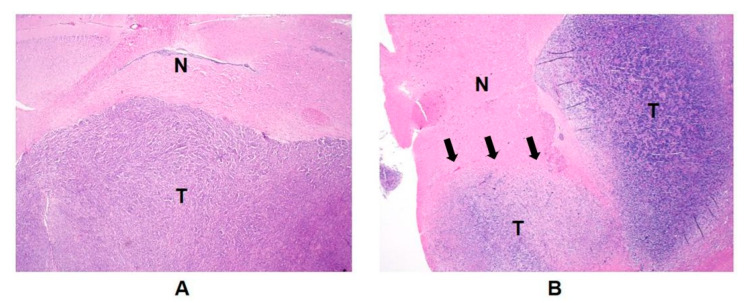
Hematoxylin and eosin (H&E) staining sections revealed a morphological difference in tumor boundaries between the enhancing (**A**) and non-enhancing tumor (**B**). While the glioblastoma model displayed a well-demarcated tumor margin without noticeable tumor cells in the nearby brain tissue (**A**), the diffuse midline glioma model exhibited an unclear tumor margin, with diffusive tumor cells appearing to infiltrate into neighboring brain tissue (**B**). T, tumor; N, normal brain tissue; magnification, ×20.

Another interesting finding from this study was that the non-enhancing tumor models exhibited a comparable level of normalized pyruvate between the ipsilateral lesion and the contralateral brain tissue, while the enhancing tumor models had a significantly smaller level of normalized pyruvate in the contralateral brain tissue compared to the ipsilateral lesion (Figure 3). In addition, the level of normalized pyruvate in the contralateral brain tissue of the enhancing tumor was significantly smaller than those in the ipsilateral lesion and the contralateral brain tissue of the non-enhancing tumor. Unlike clinical glioblastoma, which carries highly infiltrative tumor characteristics, with tumor cells extending beyond the boundary of contrast enhancement and T2 hyperintensity [21,22,23], the preclinical model of glioblastoma using the U87-MG cell line has been shown to maintain relatively well-demarcated tumor margins in anatomical MRI and pathological slides [24]. Our result from the H&E stain of the enhancing tumor was consistent with this finding, displaying a clearly delineated tumor boundary without noticeable tumor infiltration into the nearby brain tissue (Figure 4A). In contrast, the SF8628 cell line used to create the non-enhancing tumor was derived from a patient biopsy with diffuse midline glioma, and the intracranial model of diffuse midline glioma using this cell line has been shown to contain diffuse, infiltrative, and viable cancer, which recapitulated the pathologic characteristics of the diffuse midline glioma [25]. H&E staining of the non-enhancing tumor in our study showed a relatively vague tumor margin, with tumor cells infiltrating into the surrounding brain tissue (Figure 4B). Given that pyruvate is much smaller than the gadolinium chelates used as contrast agents [26], the diffusive and infiltrating nature of the cell line used to create the non-enhancing tumor may have increased the blood–brain barrier and vessel permeability to pyruvate in the non-enhancing lesion and its contralateral brain tissue, but not to contrast agents, allowing greater transport of pyruvate across the blood–brain barrier in these tissues compared to the relatively unaffected contralateral brain tissue of the enhancing lesion. Further research is warranted to elucidate the molecular and pathological basis for these imaging findings.

Currently, clinical imaging modalities for probing in vivo metabolism are limited. Positron emission tomography (PET) is one of the most widely used clinical imaging modalities for imaging in vivo metabolism. Fluorine-18 fluorodeoxyglucose (^18^F-FDG) is the most widely used radiotracer in various clinical conditions because of its ability to probe the Warburg effect in cancer. High glycolytic metabolism is characteristic of many cancers, including high grade glioma. Unfortunately, the application of FDG-PET in patients with brain tumors is limited because of high FDG uptake in normal gray matter, which makes it difficult to delineate the tumor from the surrounding grey matter. Although various novel PET radiotracers have been used for application in brain tumors [27,28,29], the high radiation dose of PET tracers often makes it difficult to use repeatedly for longitudinal imaging examinations, which are part of the clinical routine for the management of brain tumors. In addition, the simultaneous detection of multiple PET probes is almost impossible, because the energy levels of photons generated by various PET tracers is the same. In contrast, because ^13^C resonance frequencies are widely separated in hyperpolarized ^13^C MR, ^13^C substrates and their metabolites can be detected simultaneously in one scan, allowing the acquisition of metabolic information from multiple processes. 

Proton magnetic resonance spectroscopy (^1^H MRS), another imaging modality for probing in vivo metabolism, has been widely used for measuring brain metabolites. However, ^1^H MRS has low SNR and requires a long scan time. Quantifying lactate signal in ^1^H MRS poses a technical challenge, because lactate and lipid peaks overlap in the same frequency range. In addition, ^1^H MRS measures the steady-state metabolic signal, and the understanding of the steady-state ^1^H lactate signal is complicated [30]: it relies upon the amount of lactate production and clearance from tissue. In contrast, ^13^C MR metabolic imaging with hyperpolarized pyruvate allows the real-time, in vivo detection of pyruvate and its metabolic products, including lactate, with a huge gain in sensitivity and no background signals in a very short scan time. This novel imaging strategy has enabled investigations of real-time metabolic processes that are critical to various disease conditions, but are unattainable by in vivo imaging, including cancer [31,32], cardiovascular disease [33,34], diseases of the liver and kidney [35,36], inflammation [37,38], genetic disorders [39], and other neurologic conditions [40,41]. These efforts are expected to facilitate the adoption of this technology, accelerating the essential understanding of the disease mechanism, assisting in disease diagnosis, and improving the accurate assessment of treatment outcome.

A number of pre-clinical studies have explored the feasibility of using hyperpolarized ^13^C metabolic imaging for neuro-oncologic application [10,11,13,15,16]; however, most of these studies have utilized animal models with a high-grade enhancing brain tumor. To our knowledge, only a few pre-clinical studies have applied hyperpolarized ^13^C pyruvate imaging to a non-enhancing brain tumor [25,42]**,** and no efforts have been made to compare the metabolic information between enhancing and non-enhancing tumors using this technique. The characterization and comparison of hyperpolarized ^13^C imaging findings between the two components would be helpful in understanding the distinct metabolic features, and may be beneficial in understanding the underlying biology between the two lesions. 

Our current pre-clinical study warrants further investigations to support the validity of ^13^C metabolic imaging with hyperpolarized pyruvate for differential characterization of metabolic profiles between enhancing and non-enhancing brain tumors in neuro-oncology patients. ^13^C MRI with hyperpolarized pyruvate has been applied to clinical studies in healthy human brains [43], for differentiating metabolic differences between the brain tumor and normal brain [12,14], and longitudinally demonstrating aberrant metabolism in patients with progressive glioblastoma [9]. As prospective clinical trials with a larger patient population are ongoing to apply this technology to the monitoring of a response to therapy in treated neuro-oncology patients, our current work may provide a basis for characterizing and comprehending ^13^C metabolic processes in different compartments of brain lesions in these patient populations.

## 4. Materials and Methods

### 4.1. Ethical Statement

The animal study procedures were approved by the institutional animal care and use committee, and all applicable international, national, and/or institutional guidelines for the care and use of animals were followed. 

### 4.2. Cell Culture

U-87 MG human glioblastoma multiforme (GBM) cell lines were maintained as exponentially growing monolayers in a complete medium consisting of Eagle’s minimal essential medium with 10% fetal calf serum and 1% nonessential amino acids. Cells were cultured at 37 °C in a humidified atmosphere consisting of 95% air and 5% CO2. Patient-derived human diffuse midline glioma cells (SF8628) were grown as sphere cultures in EF20 medium composed with neurobasal medium (Thermo), 20 ng/mL of EGF (Peprotech, Rocky Hill, NJ, USA), 20 ng/mL of FGF (Peprotech), 2% B27 supplement (Thermo), 0.25% N2 supplement (Thermo), 3 mM of L-Glutamine (Thermo), 2 μg/mL of Heparin (Sigma), and 1% antibiotic-antimycotic (Thermo). For tumor xenografts, cells were harvested by trypsinization, washed once with Hanks’ Balanced Salt Solution, and resuspended in Hanks’ Balanced Salt Solution for tumor implantation.

### 4.3. Tumor Implantation

Two brain tumor models were created by intracranial injections of two brain tumor cell lines into the brains of six-week-old male athymic rats (rnu/rnu, homozygous, purchased from Harlan, Indianapolis, IN, USA): (1) U87-MG (*n* = 5), which represented the enhancing tumor, and (2) SF8628 (*n* = 6), which represented the non-enhancing brain tumor. The detailed procedures for the cell culture and intracranial implantation followed previously described methods [25,44]. Before the procedure, the animals were housed under aseptic conditions with filtered air and sterilized food, water, bedding, and cages. For implantation, rats were anesthetized with an intraperitoneal injection of ketamine (60 mg/kg) and xylazine (7.5 mg/kg); 10 μL of cell suspension (5 × 10^6^ cells for U-87 MG and 1 × 10^6^ cells for SF8628) were slowly injected over 1 min into the right caudate-putamen of the rat brain at a 9.6 mm depth from the bottom of the skull. All procedures were carried out under sterile conditions. 

### 4.4. Polarization Protocol

A mixture of 35 µL (approximately 45 mg) of [1-^13^C]-pyruvate (Isotec, Miamisburg, OH, USA) and 15 mM of OX63 trityl radical, along with 1.5 mM of Dotarem (Guerbet, Villepinte, France), was hyperpolarized using a HyperSense^®^ dynamic nuclear polarization polarizer (Oxford Instruments, Abingdon, UK) at 3.35 T and 1.4 K by irradiating with 94.1 GHz microwaves using methods described previously [45]. After approximately 1 h of microwave irradiation, the hyperpolarized pyruvic acid was rapidly dissolved in a saline solution with 5.96 g/L of Tris (40 mM), 4.00 g/L of NaOH (80 mM), and 0.1 mg/L of Na2 ethylenediaminetetraacetic acid. The final dissolved solution had a concentration of 80 mM of pyruvate and a pH ~7.5.

### 4.5. ^1^H and ^13^C Imaging Examination

All imaging exams were performed using a 3 Tesla clinical MRI system (Discovery MR750, GE Healthcare, Waukesha, WI, USA). The scanner was equipped with a multinuclear spectroscopy (MNS) package, a broadband radiofrequency (RF) amplifier, and a custom-built ^1^H-^13^C RF coil. The rats’ body temperatures were controlled with a heated pad located in the RF coil. Anesthesia was achieved with a continuous delivery of approximately 1.5% isoflurane via a nose cone placed at the animals’ noses. In each imaging experiment, the following data were acquired in order: (1) high-resolution axial T2WI using a fast spin-echo sequence (echo time/pulse repetition time = 60/4000 ms, 8 cm FOV, 192 × 192 matrix, 1.5 mm slice thickness, and 8 NEX); (2) compressed sensing ^13^C 3D MRSI data with the use of a double spin-echo sequence, a variable flip angle schedule, centric k-space encoding, and echo-planar readout on the *z*-axis (echo time/pulse repetition time = 140/215 ms, effective matrix size of 20 × 16 × 16, 2 × 2 × 5.4 mm spatial resolution, acquisition time = 17 s) [24] acquired at 20 s from the start of a 10-s injection of approximately 2.5 mL of hyperpolarized [1-^13^C]pyruvate through the tail vein; and (3) high-resolution axial T1WI using spin-echo sequence (echo time/pulse repetition time = 10/700 ms, 8 cm field of view, 320 × 192 matrix, 1.2 mm slice thickness, and 6 NEX) after the injection of 0.2 mmol/kg gadolinium (Gd)-diethylenetriaminepentaacetic acid (DTPA).

### 4.6. Histology Evaluation

Immediately after the MRI examination, whole brains from the animals were excised, fixed in phosphate-buffered 10% formalin, embedded in paraffin, and stained with hematoxylin and eosin (H&E) for histological examination.

### 4.7. ^13^C Data Processing and Analysis

The compressed sensing ^13^C 3D MRSI data were processed using a method described previously [24]. Briefly, the raw data were reordered to a 4D array, and a 4D Fourier transform was applied to produce a 3D spatial/spectral array of the spectra. A linear phase correction was applied in the superior and inferior direction in order to adjust the offset in k-space points. For the quantification of the ^13^C metabolites, lactate, pyruvate, and total carbon (the sum of the peak heights from lactate, pyruvate, alanine, and pyruvate-hydrate), the signals were calculated from each spectroscopic imaging voxel using the magnitude spectra. The lactate and pyruvate signals were normalized by the maximum total carbon signal in the blood vessels, which was obtained by calculating the average of two maximum total carbon signals from a highly vascular area lateral and ventral to the brain. This region contained an external carotid artery and a jugular vein. The normalized lactate and pyruvate and the ratio of lactate to pyruvate (Lac/Pyr) were compared between enhancing and non-enhancing voxels using a two-tailed, unpaired *t*-test. The enhancing voxels were defined as voxels containing larger than 70% contrast enhancement in volume from the glioblastoma models. The non-enhancing voxels were defined as voxels containing larger than 70% T2 hyperintensity in volume from the diffuse midline glioma models. In addition, the ^13^C metabolite parameters in contralateral normal-appearing brain tissue in each tumor were calculated and compared with the ipsilateral tumor in each model using a two-tailed, paired *t*-test. The results of the quantified metabolites were expressed as the mean ± standard error of the mean.

## 5. Conclusions

This study applied ^13^C MR metabolic imaging with hyperpolarized [1-^13^C]pyruvate for the differential characterization of metabolic profiles between enhancing and non-enhancing brain tumors. Distinct metabolic profiles were found between the enhancing and non-enhancing tumors, as well as their contralateral normal-appearing brain tissues. The results from this study suggest that that this technique may be useful in characterizing the functional characteristics in heterogeneous anatomical lesions, as well as normal tissue, in brain tumors.

## Figures and Tables

**Table 1:** Summary of the metabolite quantification from the two types of tumor models. U87-MG and SF8628 cell lines were used to create the intracranial models of enhancing and non-enhancing brain tumors, respectively. The values are reported as the mean ± standard error of the mean.

		Lac/Pyr	Normalized Lactate	Normalized Pyruvate
Tumor	U87-MG (*n* = 5)	1.29 ± 0.16 ^a,‡^	0.28 ± 0.06 ^b,†^	0.20 ± 0.02 ^†^
	SF8628 (*n* = 6)	0.69 ± 0.05 ^a,‡^	0.11 ± 0.02 ^b,‡^	0.16 ± 0.02
Contralateral brain tissue	U87-MG (*n* = 5)	0.37 ± 0.10 ^‡^	0.04 ± 0.01 ^†^	0.11 ± 0.01 ^c,†^
	SF8628 (*n* = 6)	0.26 ± 0.04 ^‡^	0.05 ± 0.01 ^‡^	0.18 ± 0.02 ^c^

Lac/Pyr, the ratio of lactate to pyruvate; nLac, normalized lactate; nPyr, normalized pyruvate. ^a^ *p* < 0.01 between U87-MG tumor vs. SF8628 tumor, ^b^ *p* < 0.05 between U87-MG tumor vs. SF8628 tumor, ^c^ *p* < 0.05 between U87-MG contralateral vs. SF8628 contralateral, ^‡^ *p* < 0.01 between tumor vs. contralateral within the same type of tumor cell line, ^†^ *p* < 0.05 between tumor vs. contralateral within the same type of tumor cell line.

## Data Availability

The data presented in this study may be available on request from the corresponding author.
